# AdipoRon Modulation of the Invasive Potential of Prostate Cancer Cells Is Dependent on Their EMT-Related Phenotype

**DOI:** 10.3390/biomedicines14071618

**Published:** 2026-07-17

**Authors:** Ana Luiza Romano Gabriel, Francesca Bianchi, Michele Sommariva, Stefano Vinci, Martina Anselmi, Sergio Luis Felisbino, Nicoletta Gagliano

**Affiliations:** 1 Department of Structural and Functional Biology, Institute of Biosciences, São Paulo State University (UNESP), Botucatu 18618-689, SP, Brazil; ana.l.gabriel@unesp.br (A.L.R.G.); sergio.felisbino@unesp.br (S.L.F.); 2 Department of Biomedical Sciences for Health, Università degli Studi di Milano, 20133 Milan, Italy; francesca.bianchi1@unimi.it (F.B.); michele.sommariva@unimi.it (M.S.); stefano.vinci@unimi.it (S.V.); 3 UO Laboratorio di Morfologia Umana Applicata, IRCCS Policlinico San Donato, 20097 San Donato Milanese, Italy; 4 Unit of Microenvironment and Biomarkers of Solid Tumors, Department of Experimental Oncology, Fondazione IRCCS—Istituto Nazionale dei Tumori, 20133 Milan, Italy; martina.anselmi96@gmail.com

**Keywords:** epithelial-to-mesenchymal transition, prostate cancer, AdipoRon, 3D spheroids, E-cadherin, matrix metalloproteinases

## Abstract

**Background/Objectives**: The epithelial-to-mesenchymal transition (EMT) process provides cancer cells with morpho-functional characteristics for invasion and metastasis. A reduction in serum adiponectin (APN) has been detected in prostate cancer (PCa) patients. Since AdipoRon (AdR), an agonist of APN, has been demonstrated to exert potent antitumor effects on cancer cells, including PCa, we explored the effects of AdR on PCa cells with different EMT-related traits. **Methods**: DU145 and PC3 cells were grown in 3D spheroids and treated with AdR or left untreated (CT). The expression of APN receptors, EMT markers, and invasive potential were analyzed using an integrated molecular and morphological approach. **Results**: APN receptor mRNA levels were higher in DU145 than in PC3 cells and were markedly increased only in DU145 spheroids upon AdR administration, while remaining unchanged in PC3 spheroids. AdR treatment was associated with MAPK signaling activation and modulation by AdR only in DU145 spheroids. Molecular and morphological analysis confirmed the “more epithelial” phenotype of DU145, compared with the “more mesenchymal” PC3 cells with higher N-cadherin expression, and EMT markers were not broadly affected by AdR administration in either cell line. MMP-2 activity was similar across experimental conditions, whereas MMP-9 activity was higher in PC3 than in DU145 spheroids and was not directly modulated by AdR. Although TIMP-2 mRNA levels were similar in the different experimental conditions, TIMP-1 mRNA and protein levels were higher in PC3 than in DU145 spheroids, and AdR increased TIMP-1 protein expression in PC3 cells. However, the MMP-2/TIMP-2 ratio strongly decreased in both AdR-treated DU145 and PC3 spheroids, compared with the CT, and a similar modulation for MMP-9/TIMP-1 was limited to DU145 cells. **Conclusions**: Overall, our results suggest that AdR activity can be associated with the modulation of invasion-associated molecular features of PCa spheroids, depending on their EMT-related phenotype.

## 1. Introduction

The prostate is a male reproductive accessory gland crucial for reproduction but prone to inflammatory conditions and age-related disorders, including benign prostatic hyperplasia and prostate cancer (PCa) [1,2,3]. Indeed, PCa is the second most diagnosed solid tumor in men, with an estimated 1,466,680 new cases reported in 2022 [4]. Metastatic PCa alone causes over 400,000 deaths annually, and its mortality rate is expected to more than double by 2040 [4,5,6]. Most metastatic cases eventually progress to castration-resistant prostate cancer, a stage marked by resistance to androgen deprivation therapy, limited treatment options, and rapid disease progression, making it a leading cause of PCa-related morbidity and mortality [7,8,9].

Adiponectin (APN) is an adipokine primarily secreted by adipose tissue but also produced by several other cell types, circulating in different oligomeric forms and acting through its receptors, AdipoR1 and AdipoR2 [10,11,12]. These receptors activate multiple downstream signaling pathways, including AMPK, MAPK, and PPARα/γ, improving insulin sensitivity and influencing key cellular processes such as proliferation, apoptosis, and chromatin remodeling [13,14]. Low APN levels are widely associated with cancer progression [15,16,17], and exogenous or receptor-mediated APN activity has been shown to inhibit proliferation, migration, invasion, and epithelial-to-mesenchymal transition (EMT) in different cancer models, suggesting its potential role as both a therapeutic target and biomarker [18]. The first orally active APN receptor agonist, AdipoRon (AdR), was identified in 2013 and has since demonstrated antitumor effects in several types of cancer, including PCa, by activating AMPK and p38 MAPK pathways, inducing apoptosis, and inhibiting EMT [19,20,21,22,23,24]. However, despite its promising anticancer properties, AdR has not yet been extensively investigated as a therapeutic strategy for prostate cancer, highlighting an unexplored area of research with significant potential.

EMT is a multi-step, reversible biological process in which epithelial cells lose key epithelial characteristics, such as cell polarity and adhesion, mainly through E-cadherin downregulation, and acquire mesenchymal traits such as increased motility, invasiveness, and secretion of extracellular matrix (ECM) components [25,26]. During EMT, a reorganization of the cytoskeleton can also be observed, especially involving actin filaments and intermediate filaments, with the loss of cortical actin typical of epithelial cells and the expression of vimentin and α-smooth muscle actin [26,27,28]. In cancer cells, EMT also increases local invasion, angiogenesis, and metastatic spread, with disseminated cells often reverting through mesenchymal-to-epithelial transition (MET) to support secondary tumor growth [26,28].

EMT is driven by transcription factors such as Snail, Slug, Zeb1, and Twist, which repress E-cadherin expression and induce mesenchymal traits [28,29,30,31]. Indeed, loss of E-cadherin, with concomitant expression of N-cadherin in the so-called “cadherin switch,” is considered a hallmark of EMT and strongly correlates with poor prognosis in several cancers, including PCa, where it is associated with higher Gleason scores and advanced clinical stage [30,32,33,34,35,36]. However, EMT is not an all-or-nothing process and frequently leads tumor cells to acquire a hybrid epithelial/mesenchymal phenotype, reflecting the plasticity that underlies cancer progression and metastasis [26,27,28,37].

Together with EMT, matrix metalloproteinases (MMPs) are particularly relevant in the modulation of the aggressive cancer cell phenotypes. MMPs are zinc-dependent endoproteases that degrade collagen and ECM components and regulate processes such as cell migration, proliferation, angiogenesis, immune response, and tumor progression [38]. Their dysregulation is frequently observed in several tumors and is associated with enhanced invasion, metastasis, and tumor growth, as seen in glioblastoma, ovarian, pancreatic, and prostate cancers [39,40,41,42,43]. Among them, MMP-2 and MMP-9 play key roles by degrading type IV collagen in the basement membranes—thus promoting angiogenesis and, importantly, tumor invasion and metastasis—and are considered important prognostic markers, with their overexpression linked to poor outcomes in multiple cancers [44,45,46].

Three-dimensional (3D) cell culture systems are widely recognized as reliable in vitro experimental models for studying cancer cell behavior and cancer drug discovery; they also maintain a 3D cell arrangement that mimics the in vivo situation in tissue and tumors [47,48,49,50,51,52], including in PCa [30,53]. Since 3D spheroids are a valuable model for assessing the invasive behavior of cancer cells and for characterizing EMT-related phenotypes [30,51,52], we investigated the in vitro effects of AdR, an APN agonist with anticancer activities, on the invasive potential of PCa cells exhibiting different EMT-related phenotypes.

## 2. Materials and Methods

### 2.1. Cell Cultures

The human androgen-independent prostate cancer cell lines DU145 and PC3 (American Type Culture Collection, ATCC, Manassas, VA, USA) were cultured in 3D spheroids; these are the most common type of human castration-resistant prostate cancer cells used for in vitro studies and have been well characterized in our laboratory. Cells were maintained at 37 °C in a humidified atmosphere containing 5% CO_2_, using RPMI medium supplemented with 10% heat-inactivated fetal bovine serum (FBS), 2 mM glutamine, and antibiotics (100 U/mL penicillin, 0.1 mg/mL streptomycin) (Euroclone, Pero, Milan, Italy). Cells were grown in T75 flasks, and their viability was determined using Trypan blue staining.

For 3D spheroid formation, PCa cells (5 × 10^4^ cells per well) were seeded in 1% agarose-coated 24-well multi-well plates (1 spheroid was formed in each well), then cultured for 7 days to reach a sufficient cell density and spheroid stability. Spheroid formation was evident after 3 days, and spheroid morphology and integrity were monitored every 2 days using phase-contrast microscopy. Approximately 300 spheroids were generated per condition, with average diameters of ~500 μm for DU145 and ~1 mm for PC3 cultures. For the analysis, the spheroid of each well was collected, and all spheroids were pooled in two replicates that were analyzed separately. The final result was the mean value of the two replicates. Spheroids were treated with 150 μM AdipoRon (AdR) (TargetMol, Wellesley Hills, MA, USA) dissolved in DMSO on day 7 post-seeding for 48 h [54]. Spheroids treated with AdR vehicle (0.1% DMSO) served as a control (CT). For molecular analysis, approximately 48–72 spheroids were pooled into two independent biological replicates per cell line and treatment. Biological duplicates were analyzed separately.

### 2.2. Spheroid Morphological Analysis and Cell Viability

After seeding the cells, the 3D spheroid growth was observed and imaged under a phase-contrast microscope to monitor spheroid growth and morphology. To assess cell viability, spheroids were incubated with 2 µM Calcein AM (Invitrogen, Thermo Fisher Scientific, Segrate, Italy), a membrane-permeable live-cell labeling fluorescent dye. Forty-eight hours after treatment, the cell culture medium was carefully removed, and spheroids were gently washed with PBS. A Calcein AM working solution was prepared at a final concentration of 2 μM in PBS and added to the spheroids. The multi-well plate was then incubated at 37 °C in a humidified CO_2_ incubator for 45 min. Following incubation, the Calcein AM solution was removed, and fresh complete culture medium was added. Spheroids were incubated for an additional 45 min at 37 °C. For nuclear staining, spheroids were incubated with Hoechst 33342 (Cell Signaling Technology Inc., Euroclone, Pero, Milan, Italy) at a final concentration of 5 µg/mL for 10 min at 37 °C. After staining, spheroids were fixed at room temperature in 4% paraformaldehyde in PBS supplemented with 2% sucrose for 30 min and then collected and mounted onto glass slides using Mowiol for confocal analysis.

### 2.3. Gene Expression Analysis by Real-Time PCR

Total RNA was extracted using the TriFast reagent (Euroclone, Pero, Milan, Italy) containing phenol and guanidinium thiocyanate in a monophasic solution. For each extraction, approximately 24–48 3D spheroids were used. After concentration quantification and integrity control, 1 µg of total RNA was reverse-transcribed using a commercial kit (iScript cDNA Synthesis Kit, Bio-Rad, Milan, Italy) in a final volume of 20 µL. PCR reactions were performed using a Bioer LineGene 9600 thermal cycler (Bioer, Hangzhou, China) in triplicate sample runs. Glyceraldehyde 3-phosphate dehydrogenase (GAPDH) gene expression was used as an endogenous control to normalize the differences in RNA content. Primer sequences used for amplification are listed in Table 1. The cycle threshold (Ct) was determined, and gene expression levels relative to those of GAPDH were calculated using the ΔCT method. The relative gene expression of the target gene normalized to GAPDH was obtained using the formula 2^(CT(GAPDH) − CT(target))^ = expression.

### 2.4. Immunofluorescence and Confocal Analysis

Spheroids grown in the wells of 24-well plates were washed in PBS; fixed for 30 min at room temperature in 4% paraformaldehyde in PBS containing 2% sucrose; post-fixed in 70% ethanol; and stored at −20 °C until use. After washing in PBS, spheroids were incubated with the following primary antibodies diluted in PBS: mouse anti-E-cadherin (1:500, Becton Dickinson, Milan, Italy), rabbit anti-N-cadherin (1:100, Santa Cruz Biotechnology Inc., Dallas, TX, USA), and mouse anti-vimentin (1:200, Novocastra, Leica Microsystems, Milan, Italy). Alexa Fluor 488-conjugated secondary antibodies (1:500, Molecular Probes, Invitrogen, Monza, Italy) were applied for 1 h at room temperature in PBS. To visualize the actin cytoskeleton, 25 µM rhodamine–phalloidin (Sigma-Aldrich, Milan, Italy) was added during incubation with the secondary antibody. Nuclear staining was performed using DAPI staining (1:100,000, Sigma-Aldrich, Milan, Italy) for 15 min. After washing, spheroids were finally mounted onto glass slides using Mowiol mounting medium (Sigma-Aldrich, Milan, Italy). Samples were acquired using an AX R laser scanning confocal microscope (Nikon Instruments Inc., Melville, NY, USA) with a 20× objective (NA 0.7).

### 2.5. Western Blot

3D spheroids were carefully washed in PBS, and whole-cell lysates were obtained using M-PER Mammalian Protein Extraction Reagent (Thermofisher Scientific, Segrate, Italy) containing freshly added protease inhibitors (Sigma-Aldrich, Milan, Italy) and 1 mM sodium orthovanadate to inhibit phosphatases. After a 30 min incubation on ice and vortexing, samples were centrifuged at 14,000× *g* for 15 min at 4 °C to remove cell debris. Total protein concentration was determined using a colorimetric method (DC Protein Assay, Bio-Rad Laboratories, Segrate, Italy), and 30 μg of protein per sample was mixed with sample buffer (Bio-Rad Laboratories, Segrate, Italy), separated in 10% SDS-PAGE under reducing and denaturing conditions, and transferred onto nitrocellulose membranes using a Trans-Blot Turbo system (Bio-Rad Laboratories, Segrate, Italy) according to the manufacturer’s instructions. Membranes were blocked for 10 min in EveryBlot Blocking Buffer (Bio-Rad Laboratories, Segrate, Italy) and incubated with the following primary antibodies diluted in PBST: anti-E-cadherin (1:2500, Becton Dickinson, Milan, Italy), anti-N-cadherin (1:1000, Santa Cruz Biotechnology Inc., Dallas, TX, USA), and anti-vimentin (1:1000, Leica Microsystems, Milan, Italy). Immunoreactive bands were detected using horseradish peroxidase (HRP)-conjugated secondary antibodies (Cell Signaling Technology Inc., Euroclone, Pero, Milan, Italy), and Amplified Opti-4 CN substrate (Biorad Laboratories, Segrate, Milan, Italy). To analyze the expression pattern of AKT and MAPK, the membranes were incubated with primary antibodies: anti-AKT (1:1000, Cell Signaling Technology Inc., Euroclone, Pero, Milan, Italy), pAKT (1:1000, Cell Signaling Technology Inc., Euroclone, Pero, Milan, Italy), MAPK (1:1000, Cell Signaling Technology Inc., Euroclone, Pero, Milan, Italy), and pMAPK (1:1000, Cell Signaling Technology Inc., Euroclone, Pero, Milan, Italy). This was performed o.n. at 4 °C. Immunoreactive bands were detected using HRP-conjugated secondary antibodies (Cell Signaling Technology Inc., Euroclone, Pero, Milan, Italy) and the SuperSignal West Femto Maximum Sensitivity Substrate (Thermo Scientific, Waltham, MA, USA). Twist, TIMP-1, and TIMP-2 protein levels were investigated with primary antibodies (1:1000, Cell Signaling Technology Inc., Euroclone, Pero, Milan, Italy) and SuperSignal West Femto Maximum Sensitivity Substrate (Thermo Scientific, Waltham, MA, USA). Membranes were reprobed with a monoclonal anti-α-tubulin antibody (1:2000; Sigma-Aldrich, Milan, Italy), which served as a loading control for normalization. Immunoreactive bands were analyzed using densitometric scanning (UVBand, Eppendorf, Milan, Italy).

### 2.6. SDS-Zymography

SDS-zymography cells were cultured in serum-free cell culture media for 48 h. Cell culture supernatants obtained from 3D spheroids were centrifuged to remove cell debris and mixed 3:1 with a sample buffer (containing 10% SDS). Samples (10 μg of total proteins) were run at 4 °C under non-reducing conditions on 10% SDS-PAGE co-polymerized with 1 mg/mL of type I gelatin (Sigma-Aldrich, Milan, Italy). After electrophoresis, the gels were washed in a refolding buffer containing 2.5% Triton X-100 and 50 mM Tris-HCl, pH 7.5, and incubated overnight in a substrate buffer (50 mM Tris-HCl, 5 mM CaCl_2_, NaN_3_ 0.02%, pH 7.5) at 37 °C. Finally, gels were stained with Coomassie brilliant blue R250 and destained to reveal the gelatinolytic activity of the MMPs, which appeared as clear bands on a blue background. Lysis bands were quantified by densitometric analysis (UVBand, Eppendorf, Milan, Italy).

### 2.7. Statistical Analysis

Statistical analysis was performed using the GraphPad Prism v9.3 software (GraphPad Software Inc., La Jolla, CA, USA). Data were obtained from at least two replicate experiments for each cell line in each experimental condition, cultured in duplicate, and are expressed as mean ± standard deviation (SD). One-way ANOVA followed by Tukey’s multiple comparisons post hoc test was used to compare experimental groups. *p*-values lower than 0.05 were treated as statistically significant. However, since data were derived from two replicates, due to the limited sample size, the statistical analysis should be interpreted as exploratory.

Since this study includes a small sample size, Cohen’s *d* and the small-sample bias-corrected Hedges’ *g* were used to assess the strength of group differences independently of sample size [55,56]. According to Cohen’s conventional criteria, effect size values exceeding 1.2 were considered very large, while values greater than 2.0 indicated extremely large effects. We considered the effect size for those differences between CT and AdR-treated spheroids that we found strongly influenced by AdR treatment.

## 3. Results

### 3.1. Characterization of Spheroid Morphology, Cell Viability, and Proliferation

**Figure 1:**
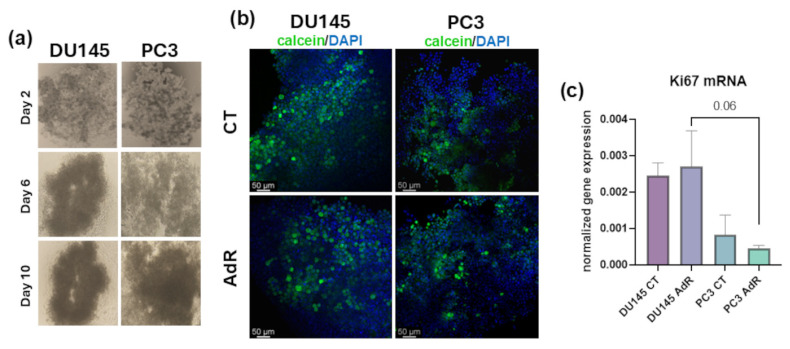
Spheroid morphology, cell viability, and proliferation. (**a**) Micrographs from the phase contrast microscope showing spheroid morphology at the indicated time points. An individual spheroid is shown in each micrograph. DU145 cells form spheroids containing densely packed cells despite an irregular profile, but PC3 spheroids are loosely packed. Original magnification: 10×. (**b**) Representative micrographs showing CT and AdR-treated spheroids upon Calcein AM incubation. Green: Calcein. Blue: Hoechst staining. Scale bar: 50 µm. (**c**) Representative bar graph showing Ki-67 mRNA levels in CT and AdR-treated spheroids. Data are expressed as mean ± SD. Due to the limited sample size, statistical analysis should be interpreted as exploratory.

Evaluation of spheroid morphology under a phase contrast microscope revealed that the DU145 cell line generated compact spheroids characterized by a regular morphology with tightly apposed cells, while spheroids containing PC3 cells were irregular, flattened, and loosely aggregated (Figure 1a), as we previously reported [30].

Similarly, Calcein AM staining revealed viable cells throughout the spheroids, both in superficial and deep regions of the CT and AdR-treated DU145 spheroids. A comparable pattern was also observed in PC3 spheroids, although fewer live cells were detectable compared with DU145 (Figure 1b).

To evaluate cell proliferation in spheroids, we analyzed the expression of the MKI67 gene, coding for Ki67, a proliferation marker expressed in cells in the active phases of the cell cycle (G1, S, G2, and mitosis) but not in resting cells (G0) [57,58]. Ki67 mRNA expression levels were higher in DU145 than in PC3 spheroids, suggesting that variable aggregation in densely and loosely packed cells, respectively, in DU145 and PC3 spheroids influences cell proliferation. Ki67 mRNA levels were not modified upon AdR administration compared with the CT, but they tended to be lower in PC3 than in DU145 (Figure 1c).

### 3.2. Gene Expression Analysis of Adiponectin Receptors

**Figure 2:**
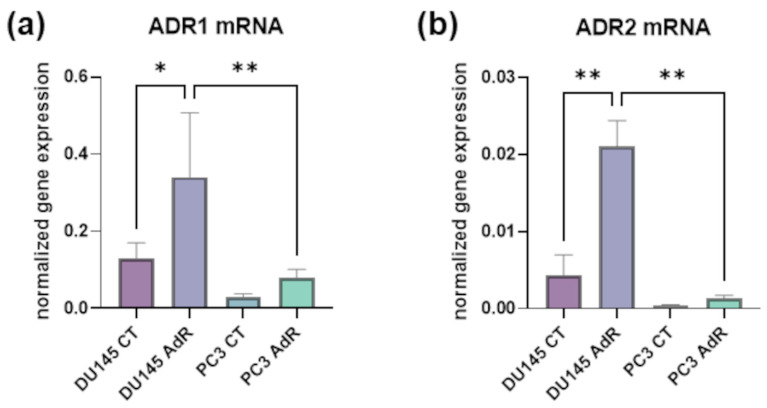
Adiponectin receptor gene expression. Bar graphs showing mRNA levels for *ADIPOR1* (ADR1) (**a**) and *ADIPOR2* (ADR2) (**b**) in CT and AdR-treated DU145 and PC3 spheroids. A modulation upon AdR treatment was evident only in DU145 spheroids. Data are expressed as mean ± SD. * *p* < 0.05; ** *p* < 0.01. Due to the limited sample size, statistical analysis should be interpreted as exploratory.

*ADIPOR1* and *ADIPOR2* mRNA levels were assessed in the CT and AR-treated DU145 and PC3 spheroids. Although *ADIPOR1* gene expression was higher than that of *ADIPOR2*, the expression patterns for both receptors were similar under the experimental conditions considered. Indeed, they were expressed to a greater extent in DU145 than in PC3 spheroids and were upregulated upon AdR treatment only in DU145 cells compared with the relative CT (*p* < 0.05 and *p* < 0.01, respectively, for *ADIPOR1* and *ADIPOR2*). Both receptors were more regulated in DU145 than in PC3 spheroids treated with AdR (*p* < 0.01) (Figure 2a,b). This result was confirmed by the Hedges’ *g* being 1,62 and 3,24, respectively, for ADR1 and ADR2 in AdR-treated vs. untreated DU145, indicating an extremely large effect.

### 3.3. Expression of AKT and MAPK

**Figure 3:**
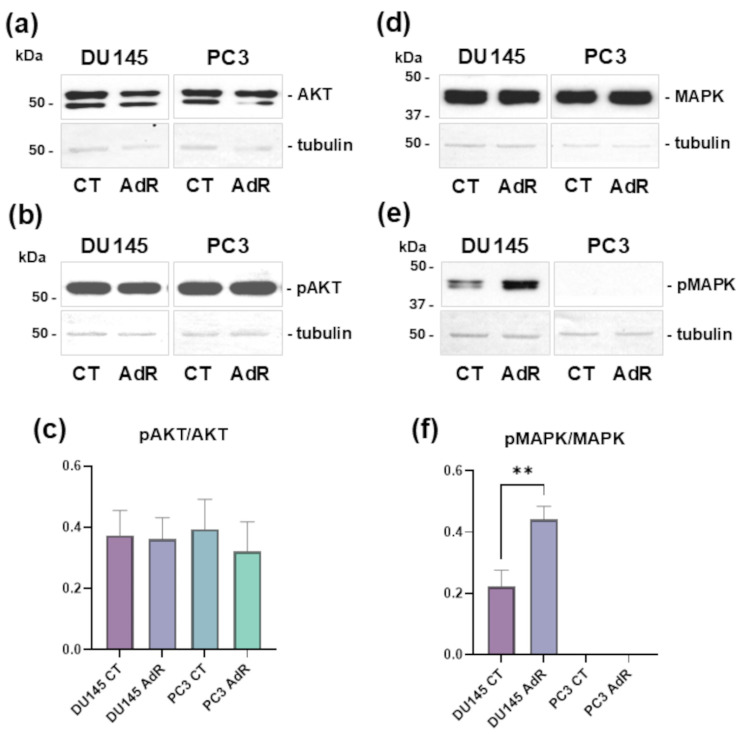
AKT and MAPK expression. Representative Western blot showing AKT/pAKT (**a**,**b**) and MAPK/pMAPK (**d**,**e**) expression in CT and AdR-treated DU145 and PC3 spheroids. Bar showing pAKT/AKT (**c**) and pMAPK/MAPK (**f**) expression after densitometric analysis of immunoreactive bands. A modulation upon AdR treatment was evident only in DU145 spheroids. Data are expressed as mean ± SD. ** *p* < 0.01. Due to the limited sample size, statistical analysis should be interpreted as exploratory.

AKT and MAPK expression and activation in the CT and AdR-treated PCa cells were assayed using Western blot. AKT and pAKT protein levels were similarly expressed in DU145 and PC3 cells, in both CT and AdR-treated spheroids (Figure 3a–c). A different pattern was observed for MAPK. Indeed, MAPK was similarly expressed in AdR-treated spheroids and CT spheroids. By contrast, pMAPK was strongly upregulated by AdR in DU145 spheroids, while it was undetectable in PC3 cells (Figure 3d,e). AdR treatment was associated with an activated MAPK pathway in DU145 spheroids, as shown in Figure 3f. This result was confirmed by the Hedges’ *g* being 2.65 in AdR-treated vs. untreated DU145, indicating an extremely large effect.

### 3.4. Expression of Epithelial Markers

Given that adiponectin has been reported to regulate EMT, we investigated whether AdR, an agonist of adiponectin receptors, similarly affects this process.

**Figure 4:**
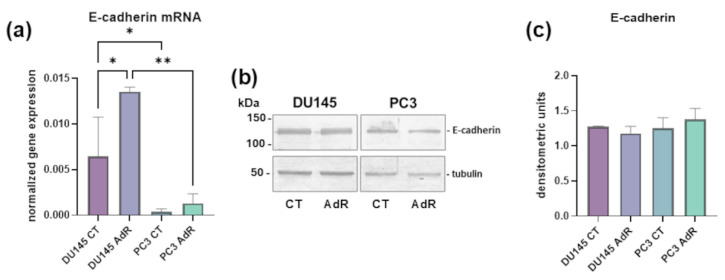
Molecular analysis of E-cadherin expression. (**a**) Bar graphs showing mRNA levels for E-cadherin in CT and AdR-treated DU145 and PC3 spheroids. Data are expressed as mean ± SD. (**b**) Representative Western blot showing E-cadherin protein levels. Membranes were reprobed with tubulin for normalization. (**c**) Bar graphs representing E-cadherin protein levels after densitometric analysis of immunoreactive bands in CT and AdR-treated DU145 and PC3 spheroids. Data are expressed as mean ± SD. * *p* < 0.05; ** *p* < 0.01. Due to the limited sample size, statistical analysis should be interpreted as exploratory.

E-cadherin expression was analyzed using molecular and morphological approaches. E-cadherin gene expression was strongly higher in untreated DU145 than in PC3 spheroids (*p* < 0.05). AdR treatment further induced E-cadherin upregulation at the mRNA level only in DU145 spheroids compared with their CT (*p* < 0.05), and this expression was also greater than in AdR-treated PC3 spheroids (*p* < 0.01) (Figure 4a). However, Western blot analysis did not confirm this pattern. Indeed, similar E-cadherin protein levels in whole-cell lysates were detected in all experimental conditions considered (Figure 4b,c).

**Figure 5:**
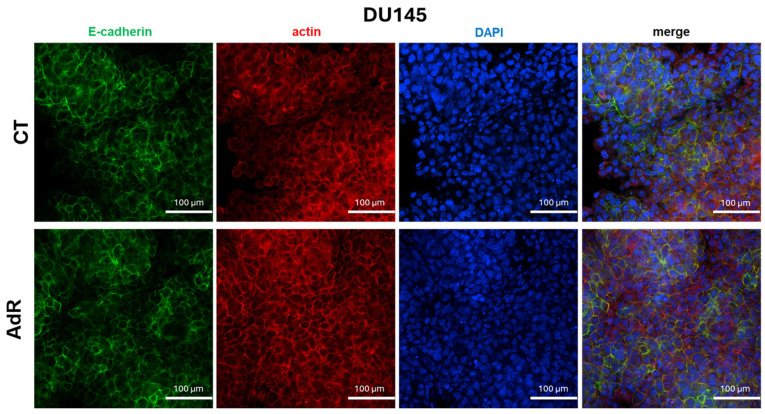
Morphological analysis of epithelial markers in DU145 spheroids. Micrographs at the confocal microscope showing E-cadherin and actin microfilaments expression and localization in CT and AdR-treated DU145 spheroids. DU145 cells exhibit a significant amount of E-cadherin at cell boundaries and actin filaments, revealing bundles of cortical actin filaments. Nuclei were stained using DAPI. Scale bar: 100 µm.

**Figure 6:**
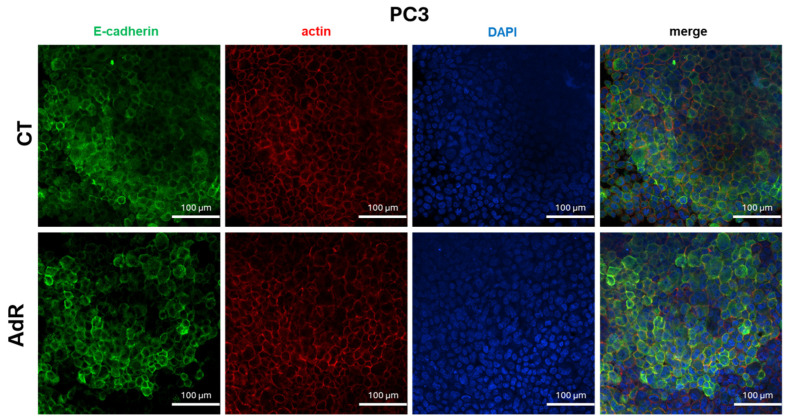
Morphological analysis of epithelial markers in PC3 spheroids. Confocal microscope micrographs show E-cadherin and actin microfilament expression and localization in CT and AdR-treated PC3 spheroids. PC3 cells have a high expression of E-cadherin at cell boundaries and evident cortical actin filaments. Nuclei were stained using DAPI. Scale bar: 100 µm.

Confocal analysis detected E-cadherin expression and localization in PCa cells. E-cadherin immunoreactivity was particularly evident at cell boundaries in CT DU145 and PC3 cells, suggesting functional cell junctions. This pattern did not seem to be influenced by AdR administration (Figure 5 and Figure 6), according to our Western blot analysis. Actin microfilaments, detected using phalloidin–rhodamine, had the same expression level in the CT and AdR-treated DU145 spheroids and the same localization observed in E-cadherin, confirming that cortical actin, typical of epithelial cells, was preserved just beneath the plasma membrane (Figure 5). Cortical actin was also detected in PC3, as in the DU145 spheroids (Figure 6).

### 3.5. Expression of Mesenchymal Markers

**Figure 7:**
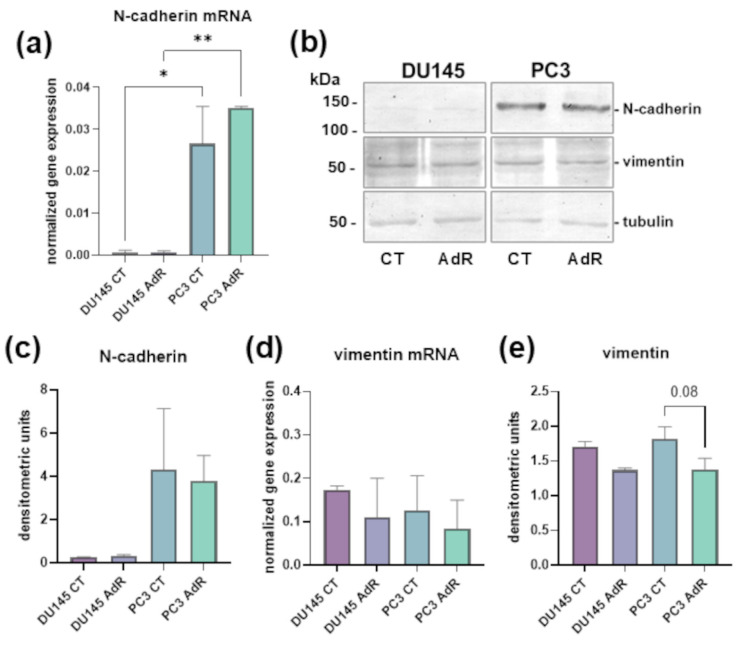
Molecular analysis of N-cadherin and vimentin expression. (**a**) Bar graphs showing mRNA levels for N-cadherin in CT and AdR-treated DU145 and PC3 spheroids. Data are expressed as mean ± SD. (**b**) Representative Western blot showing N-cadherin and vimentin protein levels. Membranes were reprobed with tubulin for normalization. Bar graphs representing N-cadherin protein levels (**c**) and vimentin gene expression (**d**) in CT and AdR-treated DU145 and PC3 spheroids. (**e**) Vimentin protein levels after densitometric scanning of immunoreactive bands in CT and AdR-treated DU145 and PC3 spheroids. Data are expressed as mean ± SD. * *p* < 0.05; ** *p* < 0.01. Due to the limited sample size, statistical analysis should be interpreted as exploratory.

Real-time PCR analysis revealed that N-cadherin gene expression was almost undetectable in DU145 spheroids but strongly expressed in PC3 spheroids. Interestingly, an evident upregulation was detected in CT PC3 compared with CT DU145 spheroids (*p* < 0.05) and in AdR PC3 compared with AdR DU145 spheroids (*p* < 0.01) (Figure 7a). A similar pattern was observed for N-cadherin protein levels in whole-cell lysates of both cell spheroids, albeit without important differences (Figure 7b,c). The mRNA levels for the mesenchymal marker vimentin were not different in the DU145 and PC3 spheroids in the CT and AR-treated samples (Figure 7d). Vimentin protein levels had a similar pattern, although they tended to decrease in AdR compared with CT PC3 spheroids (*p* = 0.08) (Figure 7b,e).

**Figure 8:**
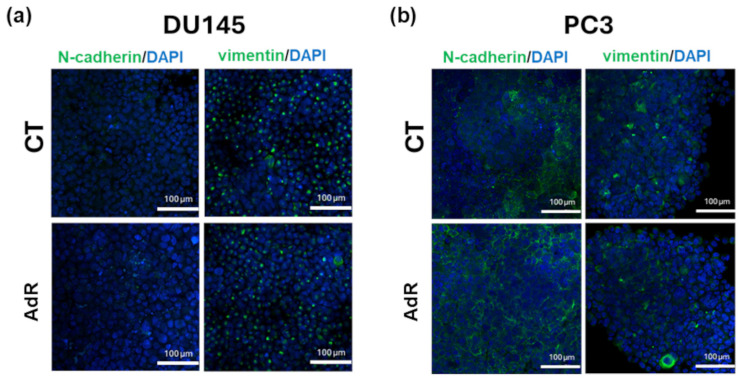
Morphological analysis of mesenchymal markers in DU145 and PC3 spheroid N-cadherin and vimentin expression. Confocal microscope micrographs showing N-cadherin and vimentin expression and localization in CT and AdR-treated DU145 (**a**) and PC3 (**b**) spheroids. N-cadherin was almost undetectable in DU145 spheroids. Nuclei were stained using DAPI. Scale bar: 100 µm.

Confocal microscopy analysis of N-cadherin and vimentin, typical mesenchymal markers, in DU145 spheroids was consistent with data obtained using molecular assays, revealing almost undetectable N-cadherin expression (Figure 8a). Furthermore, the expression of mesenchymal markers in PC3 spheroids was in line with the molecular analysis. Indeed, a similar vimentin expression pattern was detected, but strong immunoreactivity for N-cadherin was evident, showing the localization of the protein at cell boundaries with functional adhering junctions (Figure 8b). This pattern is consistent with the more epithelial and more mesenchymal hybrid phenotypes, respectively, in DU145 and PC3 cells. In both spheroids, AdR modulation of these mesenchymal markers was not detected.

### 3.6. Gene Expression for EMT Transcription Factors

**Figure 9:**
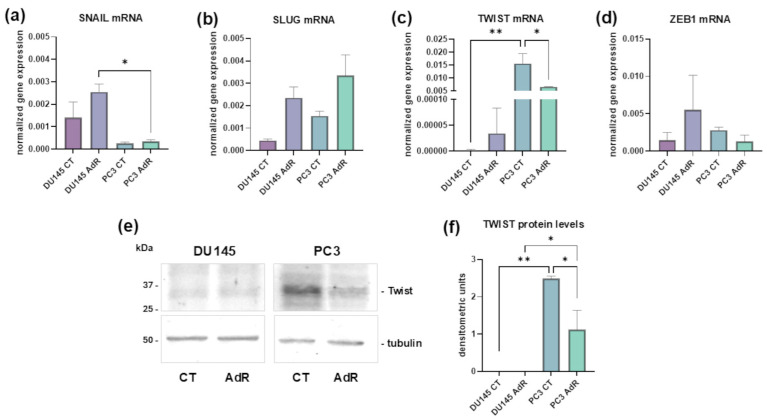
Expression of EMT transcription factors. Bar graphs showing mRNA levels for *SNAIL* (**a**), *SLUG* (**b**), *TWIST* (**c**), and *ZEB1* (**d**) in CT and AdR-treated DU145 and PC3 spheroids. TWIST expression was analyzed at the protein level using Western blot (**e**,**f**). Data are expressed as mean ± SD. * *p* < 0.05; ** *p* < 0.01. Due to the limited sample size, statistical analysis should be interpreted as exploratory.

Expression of the transcriptional regulators *SNAIL*, *SLUG*, *TWIST*, and *ZEB1* plays a key role in EMT and was analyzed at the mRNA level. We observed that *SNAIL*, *SLUG*, and *ZEB1* gene expression did not significantly differ in untreated DU145 and PC3 and was not affected upon AdR treatment (Figure 9a,b,d). However, AdR decreased *SNAIL* mRNA levels in PC3 compared to DU145 spheroids (*p* < 0.05) (Figure 9a). By contrast, *TWIST* was upregulated in CT PC3 compared with DU145 spheroids (*p* < 0.01), and its gene expression was modulated by AdR only in PC3 spheroids, showing an evident downregulation (*p* < 0.05) (Figure 9c). This pattern was confirmed with an analysis of TWIST protein levels (Figure 9e,f). The modulation of AdR on TWIST was confirmed by the Hedges’ *g* being 1.92 and 2.11 for *TWIST* mRNA and protein levels, respectively, in AdR-treated vs. untreated PC3, indicating an extremely large effect.

### 3.7. Invasive Potential

**Figure 10:**
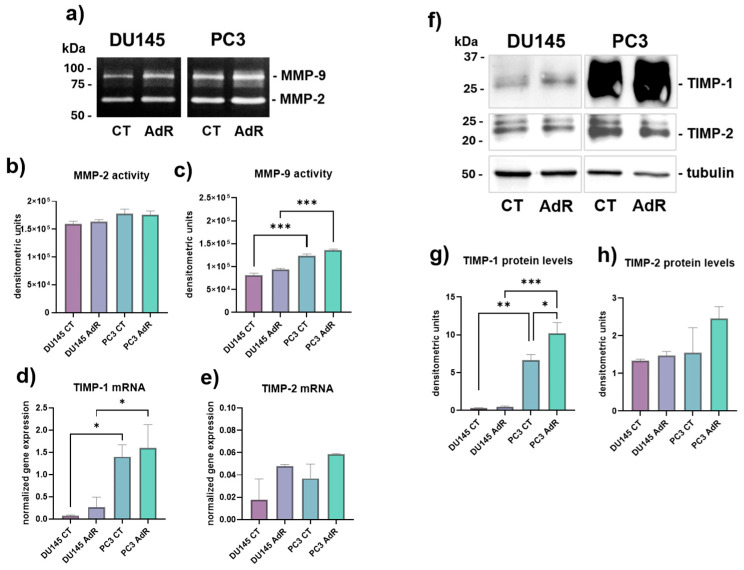
MMP and TIMP analysis. (**a**) Representative zymogram showing MMP-2 and MMP-9 lytic bands indicative of their activity. Bar graphs indicating MMP-2 (**b**) and MMP-9 (**c**) activity after densitometric analysis of lytic bands on zymograms. *TIMP-1* (**d**) and *TIMP-2* (**e**) gene expression was assayed using real-time PCR. Representative Western blot showing TIMP-1 and TIMP-2 protein levels in whole-cell lysates (**f**), as well as TIMP-1 (**g**) and TIMP-2 (**h**) protein expression. Data are mean ± SD. * *p* < 0.05; ** *p* < 0.01; *** *p* < 0.001. Due to the limited sample size, statistical analysis should be interpreted as exploratory.

Considering the relationship between EMT and tumor cell invasiveness, we evaluated the invasive potential of DU145 and PC3 spheroids upon AdR treatment by analyzing MMP-2 and MMP-9 activity in cell culture supernatants using SDS-zymography. MMP-2 activity showed no important differences in any of the experimental conditions considered (Figure 10a,b). By contrast, MMP-9 activity was higher in PC3 spheroids than in DU145 spheroids and in both CT and AdR-treated spheroids (*p* < 0.001) (Figure 10a,c).

To determine whether the activity and activation of MMPs were influenced by AdR treatment, *TIMP-1* and *TIMP-2* mRNA and protein levels were assessed. TIMP-1 expression was increased in CT PC3 compared with DU145 spheroids at the mRNA and protein levels (*p* < 0.05 and *p* < 0.01, respectively) and in AdR PC3 compared with DU145 spheroids (*p* < 0.05 and *p* < 0.001, respectively) (Figure 10d,g). Moreover, TIMP-1 expression was strongly increased upon AdR administration (Figure 10g). The modulation of AdR on TIMP-1 protein levels was confirmed by the Hedges’ *g* being 1.80 in AdR-treated vs. untreated PC3 spheroids, indicating an extremely large effect. No evident differences in TIMP-2 expression were observed for TIMP-2 (Figure 10e,h).

**Figure 11:**
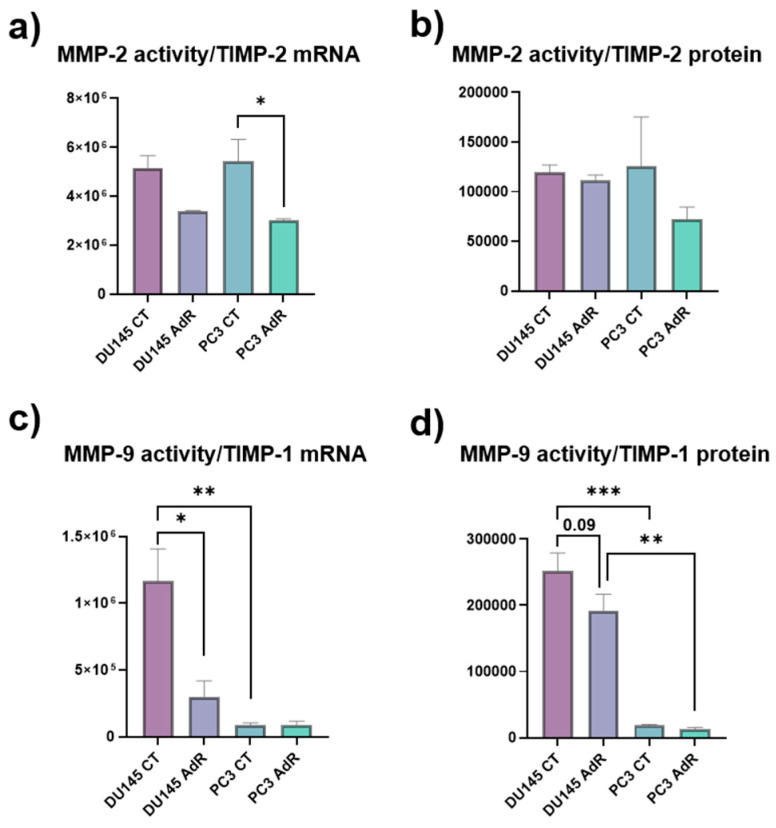
Invasive potential. To predict the invasive potential, the MMP-2 activity/TIMP-2 (**a**,**b**) and MMP-9 activity/TIMP-1 (**c**,**d**) ratios were calculated considering both TIMP mRNA and protein levels. Data are mean ± SD. * *p* < 0.05; ** *p* < 0.01; *** *p* < 0.001. Due to the limited sample size, statistical analysis should be interpreted as exploratory.

The MMP/TIMP ratio was used as an indirect functional index of the invasion-associated proteolytic balance in our experimental setting. Indeed, this ratio is intended as a functional index. The MMP-2 activity/TIMP-2 mRNA ratio decreased in PC3 spheroids (*p* < 0.05) and showed a similar trend in DU145 spheroids (*p* = 0.07) upon AdR treatment (Figure 11a), although the effect of AdR was less evident when considering the MMP-2 activity/TIMP-2 protein levels (Figure 11b). By contrast, the MMP-9 activity/TIMP-1 mRNA ratio was strongly reduced by AdR in DU145 spheroids (*p* < 0.05). This ratio was also very low in CT PC3 compared with DU145 spheroids (*p* < 0.01) but was unaffected by AdR treatment in these cells (Figure 11c). A similar pattern was observed for the MMP-9 activity/TIMP-1 protein levels (Figure 11d). Indeed, the downregulation of the MMP-9/TIMP-1 protein levels was confirmed by the Hedges’ *g* being 1.32 in AdR-treated vs. untreated DU145, indicating an extremely large effect.

## 4. Discussion

Many studies have demonstrated that 3D spatial organization captures the complex interplay of cell–cell interactions. Three-dimensional cultures can also mimic the architecture of living tissues, providing a reliable platform for studying cancer cell biology and the effect of therapeutic approaches [47,48,49,50,51,52,53,59]. Given previous evidence showing that AdR modulates PCa cell behavior [54,60], we investigated its impacts on the expression of EMT-related markers and the invasive potential of DU145 and PC3 spheroids. Moreover, we explored whether AdR effects depend on the EMT-related intrinsic phenotype of PCa cells.

As previously reported, both DU145 and PC3 cells have hybrid traits, but DU145 cells exhibit a “more epithelial” phenotype than the PC3 cell line, which displays “more mesenchymal” characteristics [30]. Accordingly, spheroids containing DU145 cells were more regular and densely packed, whereas those composed of PC3 cells grew as loosely packed aggregates. However, Calcein staining indicated preserved cell viability and did not reveal obvious large necrotic regions under the analyzed conditions. Proliferation was assessed only at the transcriptional level through *MKI67* mRNA expression. AdR treatment did not alter spheroid morphology or Calcein fluorescence, indicating that this compound does not exert any direct cytostatic or pro-apoptotic effects on PCa cells. This observation is consistent with previous studies demonstrating that AdR does not affect proliferation or apoptosis in PCa cells [60], although the opposite effect was reported in other cancer cell types [61]. Thus, our findings support the notion that AdR-mediated modulation of cell viability is cell-type dependent. This conclusion is reinforced by the analysis of Ki67 gene expression, which revealed comparable mRNA levels between control and AdR-treated spheroids, indicating a similar proliferative capacity under both experimental conditions.

AdR exerts its effects through AdipoR1 and AdipoR2, the APN receptors most associated with its beneficial activity in tissues [62]. Binding APN to its receptors activates distinct signaling pathways: AdipoR1 primarily stimulates AMPK signaling, a key regulator of cellular energy homeostasis, whereas AdipoR2 predominantly induces PPAR activation, which regulates metabolic functions [63]. Reduced expression of both receptors in PCa tissue compared with benign prostate tissue has been reported [64], suggesting that the APN signaling axis could play an important role in cancer initiation and progression. AdipoR expression has also been demonstrated in PCa cell lines [65,66], and AdipoR1 was found to be more strongly expressed than AdipoR2 in PC3 cells [65]. Similarly, our results show that DU145 and PC3 cells grown in 3D spheroids express AdipoR1 and AdipoR2 to different extents, with AdipoR1 more abundant than AdipoR2 in both cell lines. Interestingly, AdipoR expression levels were higher in DU145 than in PC3 spheroids; AdR treatment upregulated both receptors exclusively in DU145 and not in PC3 spheroids, which instead exhibited a lower gene expression level compared with AdR-treated DU145 spheroids. This finding is consistent with the hypothesis that PCa cells with distinct EMT-related phenotypes exhibit different AdipoR levels and that their response to AdR treatment may be influenced by intrinsic cell-line-specific characteristics. Further experiments involving a broader panel of PCa cell lines will be required to confirm this relationship. Notably, since AdipoR1 is reduced in breast and hepatic cancers, and its depletion has been associated with EMT induction [67,68], its upregulation following AdR treatment in PCa cells could represent a beneficial effect that may help limit tumor progression.

Since the AKT and MAPK signaling pathways play key roles in cancer progression, including PCa [69,70], we investigated whether they were modulated by AdR. While AKT was similarly expressed in both the CT and AdR-treated DU145 and PC3 cells, the MAPK expression pattern was cell-dependent. Indeed, DU145 had higher levels of MAPK compared with PC3, which was similarly expressed in the CT and AdR-treated spheroids. Interestingly, pMAPK was detectable only in DU145 spheroids, with a strong upregulation upon AdR administration. This finding suggests that MAPK signaling could be an AdR-associated but cell-dependent effect. This hypothesis is strengthened by the observation that pMAPK expression and upregulation were detected only in DU145 spheroids and paralleled the AdipoR1 and AdipoR2 mRNA levels. Due to the complexity of regulation and interaction of these pathways, further detailed studies need to be planned in future.

Previous studies have investigated the effect of APN and its receptor agonist AdR on EMT markers in various cancer cell types, including PCa cells. APN levels have been inversely correlated with cancer progression largely through inhibition of the EMT process across different tumor models [17,18,45,67,71,72]. However, this association cannot be generalized as, in some contexts (such as colorectal cancer), APN does not affect the expression of key EMT markers such as E-cadherin and vimentin [73]. In the present study, we specifically examined the effect of AdR on PCa cells grown as 3D spheroids, and we observed that AdR did not seem to modulate EMT markers. Interestingly, “epithelial” markers appeared to be unaltered at the protein level following AdR exposure in DU145 spheroids, as shown by protein and confocal analyses. A similar pattern was observed in PC3 spheroids.

In this context, it is important to consider that E-cadherin plays a multifaceted role in carcinoma progression, as its increase is usually interpreted as a reversion of the EMT process; however, it may also be detrimental by promoting cell seeding and adhesion of metastatic cells, as well as facilitating collective migration [74]. Accordingly, maintaining E-cadherin expression after AdR treatment could have a beneficial effect. Although E-cadherin mRNA increased in AdR-treated DU145 spheroids, this was not mirrored by changes in protein abundance or localization. Therefore, this transcriptional modulation should not be interpreted as evidence of a functional epithelial shift.

When considering these “mesenchymal” markers, we observed that neither N-cadherin nor vimentin was affected at the molecular or morphological level upon AdR treatment. These findings indicate that AdR did not significantly influence the EMT program in DU145 and PC3 cells, further supporting the hypothesis that modulation of the EMT-related phenotype may be cell-type-dependent across different cancers.

EMT is driven by transcription factors such as *SNAIL*, *SLUG*, *ZEB1*, and *TWIST* [26,28,75]. *SNAIL* is a master regulator of EMT and promotes metastatic potential by inducing the expression of mesenchymal markers [27,28,75,76,77], and its overexpression has been detected in several cancer types, including PCa [78]. Here, we show that *SNAIL* expression is higher in DU145 than in PC3 spheroids, and this difference in gene expression becomes more pronounced following AdR treatment. Interestingly, although *SNAIL* acts as an E-cadherin repressor, we observed comparable E-cadherin protein levels despite the evident differences in *SNAIL* gene expression between DU145 and PC3 spheroids. This discrepancy may be due to differences in *SNAIL* mRNA stability or post-translational modifications, as previously suggested [30], or possibly to additional cellular functions of *SNAIL* that are independent of EMT induction, such as cell survival and resistance to cell death [77,78,79]. Furthermore, only nuclear localization has been shown to reflect the active form of *SNAIL* [80], suggesting that mRNA expression levels may not accurately represent its functional activity. Although our results indicate that AdR does not significantly affect *SNAIL* gene expression, further experiments are needed to clarify the differences observed in our experimental setting.

Regarding the other EMT-associated transcription factors examined, while *SLUG* and *ZEB1* remained unchanged, *TWIST* mRNA levels were strongly higher in PC3 than DU145 spheroids. *TWIST* is another key EMT modulator that promotes the expression of mesenchymal markers such as N-cadherin [81,82], in line with our observations of its overexpression in PC3 spheroids, which exhibited the highest N-cadherin levels. Interestingly, TWIST-induced acquisition of mesenchymal traits appears to occur independently of E-cadherin expression [82], as also observed in our experimental setting, where E-cadherin and N-cadherin were concomitantly expressed in PC3 cells.

An important functional role of *TWIST* in several epithelial cancers is its ability to enhance the intravasation step of metastasis, suggesting that *TWIST* could serve as a useful prognostic biomarker in urologic cancers and a potential therapeutic target [83]. Notably, we observed a strong downregulation of *TWIST* mRNA and protein expression associated with AdR treatment in PC3 spheroids, suggesting that AdR may modulate selected EMT-related regulatory features in these cells, potentially linked to tumor aggressiveness.

Finally, the different expression patterns observed for *SNAIL* and *TWIST* in DU145 and PC3 cells, which display distinct phenotypic characteristics, are consistent with the suggestion that *SNAIL* is primarily involved in the early stages of invasion, whereas *TWIST* contributes to the maintenance of the invasive properties of cancer cells [75,84].

EMT provides carcinoma cells with the ability to invade surrounding tissues, ultimately leading to the formation of distant metastases. The invasive potential of cancer cells depends on basement membrane degradation, which facilitates their dissemination. This process largely relies on the activity of MMPs with gelatinolytic activity, particularly MMP-2 and -9 [85]. Our results show distinct activity patterns for these gelatinases. Indeed, MMP-2 gelatinolytic activity was similar in DU145 and PC3 spheroids and remained unaffected by AdR treatment. In contrast, MMP-9 activity, although higher in PC3 than in DU145 spheroids, was not influenced by AdR exposure. To interpret this finding, it is important to note that MMP activity is balanced by TIMPs that bind MMPs in a 1:1 stoichiometric ratio, thereby inhibiting their activation and activity [86]. Accordingly, we also analyzed TIMP expression, considering that TIMP-1 acts as the primary inhibitor of MMP-9, while TIMP-2 is the main inhibitor of MMP-2 [87]. Although we observed an evident AdR-associated modulation of TIMPs only for TIMP-1 protein levels, we identified different TIMP expression patterns in DU145 and PC3 cells. Therefore, we evaluated the MMP/TIMP ratio—used as an indirect functional index of the invasion-associated proteolytic balance—to better predict the effective invasive potential under our experimental setting. This approach has been previously reported to describe the functional imbalance of the MMP/TIMP axis in different conditions [88,89,90,91].

The MMP-2 activity/TIMP-2 mRNA ratio was similar in both cell lines and, interestingly, markedly decreased upon AdR treatment. Specifically, TIMP-2 has been shown to strongly suppress PCa cell growth in vivo by modulating MMP-2 activity, thereby impairing its function and inhibiting tumor cell invasion and proliferation [92]. Moreover, TIMP-1 mRNA levels were strongly higher in PC3 spheroids than in DU145 under both CT and AdR treated conditions and were strongly upregulated by AdR at the protein level in PC3 cells. The MMP-9 activity/TIMP-1 mRNA ratio was strongly decreased by AdR only in DU145 spheroids while remaining unchanged in PC3 spheroids. These findings suggest a cell-type-dependent differential modulation of the MMP/TIMP axis by AdR. In this scenario, the peculiar differences observed between DU145 and PC3 spheroids in terms of invasive potential-associated characteristics, consistent with their divergent biological behaviors, should be carefully considered, given that PCa accounts for over 90% of metastases in bone [93]. In this regard, several studies have shown that elevated MMP-9 levels, particularly in the late or advanced stages of PCa, contribute to bone metastasis formation and osteolysis [94,95]. During this process, PCa skeletal metastases are typically described as osteoblastic or mixed lytic/blastic lesions, characterized by the deposition of dense sclerotic bone [96]. Interestingly, lesions generated by PC3 cells lack osteoblastic activity, suggesting a distinct behavior for these cells. This observation is consistent with the lower MMP-9 activity/TIMP-1 ratio in PC3 compared with DU145 spheroids and with the absence of its modulation by AdR. Since a previous study reported a relationship between PCa cell invasion and ferroptosis [97], this mechanism could also be investigated in future studies aiming to characterize the modulation of PCa cells by AdipoRon.

Taken together, our findings suggest that AdipoRon does not overtly modulate the EMT program in PCa spheroids, yet it may exert subtle, cell-type-dependent effects on receptor expression and invasive potential-related characteristics. These results underscore the complexity of APN receptor signaling in prostate cancer and support further investigation into its potential as a context-specific modulator of tumor progression. This should also be considered in light of a recent study that suggested the recognition of androgen and its derivatives by membrane GPCRs [98].

The limitations of this study should be acknowledged. First, the number of biological replicates was limited; therefore, the statistical analyses should be considered exploratory. The calculation of Cohen’s *d* and the small-sample bias-corrected Hedges’ *g* was included to provide standardized estimates of the magnitude of selected group differences, but it does not overcome the limited statistical power associated with the small sample size.

Moreover, although SDS-zymography is a functional assay of MMP enzymatic activity able to test gelatinolytic degradation of the substrate needed during invasion and the MMPs/TIMPs ratio is frequently used as a functional index highly informative of the effective invasive potential, a direct invasion assay could strengthen our results. Finally, considering our data suggesting a role for MAPK signaling in our experimental setting, the modulation of MAPK signaling by specific inhibitors or silencing, as well as a dose–response analysis, could add new mechanistic insights on the differential effect of AdR on PCa cells having different EMT-related phenotypes. In this view, the analysis of a wider number of PCa cells exhibiting different phenotypic traits will be needed to extend these findings.

## 5. Conclusions

Overall, our results confirm previously reported differences between DU145 and PC3 cells grown as 3D spheroids and suggest that AdR’s effect is not associated with broad modulation of EMT markers. However, AdR differentially influenced AdipoR expression, MAPK phosphorylation, and MMP/TIMP balance in a cell type-dependent manner. Our findings highlight a possible role of the MMP/TIMP axis as a candidate effector that can be influenced by AdR to modulate the invasive-related properties of PCa cells, but they require further validation. Although EMT markers are not broadly affected by AdR treatment, the modulation of their invasive-associated characteristics seems to be dependent on the EMT-related phenotype of cancer cells, suggesting that their intrinsic traits remain an important determinant in predicting the ability of AdR to inhibit cell aspects related to tumor invasion and metastasis.

These results offer new insights into PCa biology and could be useful in the development of new therapeutic tools. Further studies of different PCa cells will be needed to validate our results and strengthen their translational relevance.

## Figures and Tables

**Table 1:** List of primers used for gene expression analysis using real-time PCR.

	Primer Forward	Primer Reverse
GAPDH	CCCTTCATTGACCTCAACTACATG	TGGGATTTCCATTGATGACAAGC
E-cadherin	GAACGCATTGCCACATACAC	GAATTCGGGCTTGTTGTCAT
N-cadherin	TGTTTGACTATGAAGGCAGTGG	TCAGTCATCACCTCCACCAT
Snail	CTTCCAGCAGCCCTACGAC	CGGTGGGGTTGAGGATCT
Slug	TGTTTGCAAGATCTGCGGC	TGCAGTCAGGGCAAGAAAAA
Twist	TGAGCAAGATTCAGACCCTCA	ATCCTCCAGACCGAGAAGG
Zeb1	GCCAATAAGCAAACGATTCTG	TTTGGCTGGATCACTTTCAAG
vimentin	TGGTCCTACCCACGCAGATT	GGCCAACCCAGAAGTTGGAA
TIMP-1	GGCTTCTGGCATCCTGTTGTTG	AAGGTGGTCTGGTTGACTTCTGG
TIMP-2	TGGAAACGACATTTATGGCAACCC	CTCCAACGTCCAGCGAGACC
AdipoR1	ACCCAAAGCTGAAGAAGAGCA	TATGGGATGACCCTCCAACG
AdipoR2	TGGAGCCCATTTTAGAGGCA	CGACCTTCCCATACCTTACAAAC
MKI67	CGTCCCAGTGGAAGAGTTGT	CGACCCCGCTCCTTTTGATA

## Data Availability

All data generated or analyzed during this study are included in the published article.

## Abbreviations

The following abbreviations are used in this manuscript:

AdipoR	Adiponectin receptor
APN	Adiponectin
AdR	AdipoRon
ECM	Extracellular matrix
EMT	Epithelial-to-mesenchymal transition
MET	Mesenchymal-to-epithelial transition
MMPs	Matrix metalloproteinases
PCa	Prostate cancer
TIMPs	Tissue inhibitors of matrix metalloproteinases
